# Evaluation of Knowledge and Attitude of Parents of Attention Deficit/Hyperactivity Disorder Children towards Attention Deficit/Hyperactivity Disorder in Clinical Samples

**Published:** 2017-01

**Authors:** Nasrin Dodangi, Roshanak Vameghi, Nastaran Habibi

**Affiliations:** Neurorehabilitation Research Center, University of Social Welfare and Rehabilitation Sciences, Tehran, Iran.

**Keywords:** *Attention Deficit/Hyperactivity Disorder*, *Knowledge and Attitude*, *Parents*

## Abstract

**Objective:** Knowledge and attitude of parents about attention deficit/hyperactivity disorder (ADHD) is an important factor in management of the disorder in children. This study investigates the parents’ knowledge and attitude towards ADHD, its symptoms, diagnosis, treatment and prognosis.

**Method:** In this cross-sectional descriptive study, the subjects were 150 parents (120 mother and 30 father) of ADHD children who were referred to a child psychiatry clinic affiliated in university of social welfare and rehabilitation sciences in Tehran. The diagnosis was made by a child psychiatrist according to DSM-IV TR criteria. The parents completed a 40 items questionnaire that was prepared by the authors and assessed their knowledge and attitude towards ADHD and source of their information.

**Results:** The most common source of parent’s information about ADHD was TV. The parent’s knowledge about the symptoms of the disorder was relatively good. But in regard to diagnosis, treatment and prognosis of the disorder, they have very low knowledge and even incorrect beliefs. The parent’s knowledge significantly correlated with their educational level (p=0.01).

**Conclusion**: In general, knowledge of the parents was low and it can lead to misdiagnosis or mismanagement of this common and important disorder and need to further consideration in terms of educating parents about the disorder in media specially TV.

Attention Deficit Hyperactivity Disorder (ADHD) is one of the most common problems among the children who are referred to mental health facilities (1). This disorder is identified with attention deficit, hyperactivity and impulsivity, and it begins chronically from childhood and often continues to adulthood (2, 3). This disorder affects various aspects of the child’s life such as educational status, social functioning and parent-child relationship. It has been found that the long-term outcome of the disorder depends on duration and level of commitment to the interventions (4).

 It is estimated that about 5 to 10% of all the school-age children are diagnosed with ADHD (5). Treatment of this disorder is primarily by stimulant medications and behavioral interventions that can be used alone or in combination with each other (6). Parents often have an ambivalent view about the interventions (7) and a poor commitment to the recommended medical interventions for this disorder (8) where the rate is estimated to be approximately 25 to 50%, which decreases over time (9). 

In a study, the commitment of families with an ADHD child was compared in three groups: One group receiving methylphenidate alone, the second, receiving methylphenidate and behavioral interventions, and the third group using placebo and behavioral interventions. Of these three groups of families, 20% discontinued medication within four months and 45% within 10 months and 51% did not complete their behavioral intervention plans (10). Lack of commitment to ADHD treatment cannot merely be justified by factors such as socioeconomic status, parental stress or family coping style. 

Another explanation for this problem is the parents’ knowledge about this disorder and their attitude toward treatment, as it has been seen that those parents who had more knowledge about the disorder used pharmacologic and non-pharmacologic therapies more than the others (11).

## Materials and Methods

This was a cross-sectional descriptive study conducted to determine the level of knowledge and attitude of parents with a child with ADHD towards this disorder.

First, a questionnaire was prepared using the relevant sources and available articles as well as the tools previously made to assess the knowledge and attitude of different respondents to this disorder, and its validity and reliability was assessed.

After analyzing the results and applying the necessary changes, the intended questionnaire was finalized and ready to use. Then the prepared questionnaires were provided to the parents of children with ADHD.

***Participants***

The study participants were the parents of 6 to 12 year old children with ADHD who referred to Akhavan and Rofayde Child and Adolescent Psychiatric Clinics in Tehran.

***Sampling Method***

In this study, samples were gradually collected through convenience sampling, meaning that each of the individuals who referred to the aforementioned clinics was entered into the study in case of having the inclusion criteria and providing consent.

***Instruments***

1. Demographic Questionnaire (author made): This questionnaire included age and gender of the child, age and education of the parents.
2. The Kiddie-schedule for Affective Disorders and Schizophrenia (K-SADS): This semi-structured interview allows the interviewer to make judgment during the interview (12). The reliability and validity of the Persian version of K-SADS has been established in Iran previously (13, 14).
3. The Knowledge and Attitude Questionnaire: It is a 40-item questionnaire, designed by the researcher, and includes the source or sources of information of parents about ADHD, their knowledge about the symptoms and their attitudes toward the cause, prognosis, treatment and possible side effects of the therapy.

Given that this questionnaire was not already present in a standard form, it was prepared as follows:

***Questionnaire Compilation Method***

After collecting the survey questionnaires, content and statistical analysis was performed on the opinions, and the necessary changes were applied based on the results.

The next step was the pilot study. The edited questionnaire was given to 15 parents of the target population. The purpose of this step was to determine the potential cultural and linguistic ambiguities and inconsistencies of the questionnaire for the audience and to identify the implementation problems. Then the necessary modifications were made to the questionnaire.

The final step in compiling the questionnaire was to test its reliability. Cronbach’s alpha method was used to determine the reliability of the questionnaire wherethe value was calculated to be 0.667, indicating acceptable reliability.

***Statistical Analysis***

The data and the variables were entered into SPSS 18 for statistical analysis. Spearman test was used to evaluate the possible relationship between the demographic factors such as gender and parents’ education level and awareness and attitude. The significance level was set at p ≤ 0.05.

***Ethics***

Written consent was obtained from the parents, and adequate explanation was provided about the study while handing out the questionnaires.

## Results

**Figure1:**
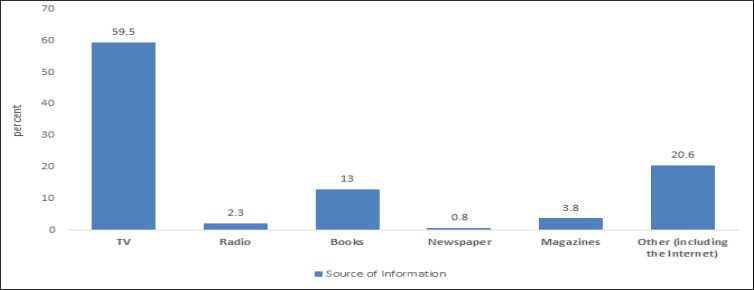
Parents’ Sources of Information about Attention Deficit/Hyperactivity Disorder

The sample included 150 children aged 6-13 years and their parents. Boys constituted 50% of the sample. The average age of children was 9±1.9 years, and 80% of the parents were mothers. Age range of the parents was 25-64 years and the age mode was 33-44 years old (52.1%). Parents’ level of education ranged from elementary education to doctorate. The highest and lowest frequencies were related to diploma (45.1%) and doctorate (0.7%), respectively. In 48.6% of the children, more than 6 months had passed since the diagnosis of ADHD. Among the parents, 88% were familiar with this disorder before participating in the study. TV was the main source of information for the parents (52%). Other sources are shown in Figure 1.

In 82.2% of the cases, the teacher had complained to the parents about hyperactivity or inattention of the child. In 74.5% of the cases, children had gone to the physician before participating in the study because of symptoms of hyperactivity. Table 1 shows the rate of false beliefs. 40% of parents believed that Children with ADHD were always hyperactive and 71.3% of them believed that Hyperactivity released the energy of these children and reduced their symptoms.

46% of parents reported that Heredity had no role in this disorder, 38.7% of them reported that ADHD resulted from bad parenting practices and 38% of them reported that symptoms of the disorder resulted from the IQ score. 28% said that ADHD disappeared as the child growing up. More than 45% of parents thought that if these children behaved inappropriately, they couldn’t be punished like other children. More than 52% believed that psychological testing was necessary to diagnose this disorder. 11.3% of parents reported that ADHD could be controlled by herbal medicines and 26% of them believed that Ritalin had serious side effects.

Table 2 demonstrates the rate of true beliefs about ADHD. More than 66% of parents agreed that if ADHD was not properly treated, other problems could arise, 51.3% of them said that the risk for delinquency is higher in these children and 58% reported that the risk of addiction and other psychiatric problems is higher in children with ADHD. 60% believed that these children could be as successful in their lives as others. 45.3% of them agreed that the symptoms of ADHD could be control by drugs.

In 64% of the cases, Ritalin was prescribed for these children, among whom 97.8% had taken the drug and 83.1% were satisfied with its effect. Table 3 shows parents’ level of awareness of ADHD symptoms. Parents’ level of awareness of poor attention and distractibility in ADHD was 84.7%, difficulty in doing or finishing homework was about 86%, difficulty in organizing tasks and activities was about 80% and reluctance to do homework was 84.7%. More than 92% of parents believed that children with ADHD need to monitoring them while doing their homework and 88% of them said that these children only pay attention to what they were highly interested in. 86% of parents said that ADHD associated with hyperactivity and 90% said that these children were restless and fidgety. More than 86% of them reported that children with this disorder were unable to sit still, about 80% believed that they had difficulty in waiting their turn and 73.3% reported that they were unable to play quietly.

**Figure2:**
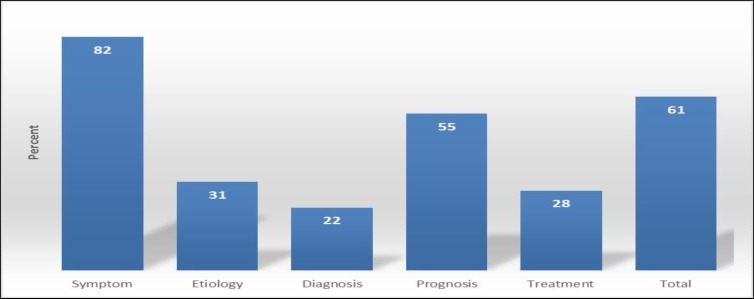
Distribution of Parents’ Level of Awareness of the Symptoms, Diagnosis, Treatment, and Prognosis of Attention Deficit/Hyperactivity Disorder

Given that the items of the questionnaire assessed parents’ level of awareness in various areas such as symptoms, diagnosis, treatment, and prognosis, the subjects’ scores in each are calculated and shown in diagrams (Figure 2).

As the diagram displays, the parents’ lowest level of awareness was related to the diagnosis and treatment of the disorder.

The Spearman’s correlation test showed that parents’ awareness of and attitude toward ADHD are associated with their level of education. In other words, the higher the education level, the higher the level of awareness (p = 0.01).

Moreover, it was found that level of education had no relationship with the level of awareness about the symptoms, etiology and diagnosis of the disorder.

However, level of education had a significant relationship with the level of awareness of prognosis and treatment of the disorder. In other words, the higher the level of education, the higher the level of awareness will be (p < 0.005 and p < 0.001, respectively).

## Discussion

The results revealed that most parents do not have enough information about the disorder. However, awareness about the symptoms of the disorder was relatively good. The rates of correct answers about the symptoms ranged from 73.3% to 92.7%. However, about one third of parents did not believe in the existence of this disorder in their children. More than two-thirds of parents believed that this disorder always accompanies hyperactivity, andthis attitude leads to ignore ADHD, predominantly inattentive type. Parental knowledge of the etiology, diagnosis, prognosis, management and treatment of this disorder was lower than their knowledge of the symptoms.

**Table1:** Distribution of Parents’ Rate of False Beliefs about Attention Deficit/Hyperactivity Disorder

**Mistaken Belief**	**Rate of False Belief (%)**
Children with ADHD are always hyperactive.	40%
Hyperactivity releases the energy of children with ADHD and reduces their symptoms.	71.3%
Heredity has no role in this disorder.	46%
This disorder results from improper upbringing by parents.	38.7%
Symptoms of ADHD result from the IQ score.	38%
Symptoms of ADHD disappear as the child grows up.	28%
If these children behave inappropriately, they can’t be punished like other children.	45.3%
Psychological testing is necessary to diagnose this disorder.	52.7%
Symptoms of ADHD can be controlled by herbal medicines.	11.3%
Ritalin has serious side effects.	26%

**Table2:** Distribution of Parents’ Rate of True Beliefs about Attention Deficit/Hyperactivity Disorder

**True Belief**	**Rate of true Belief (%)**
If ADHD is not properly treated, other problems can arise	66.7%
The risk for delinquency is higher in children with ADHD than in others	51.3%
These children can be as successful in their lives as others	60%
The risk of addiction and other psychiatric problems is higher in these children	58%
The symptoms of ADHD can be control by drugs	45.3%

**Table3:** Distribution of Parents’ Level of Awareness of Attention Deficit/Hyperactivity Disorder Symptoms

**Symptom**	**Level of Awareness of the Symptom**
Poor attention and distractibility	84.7%
Difficulty in doing or finishing their homework	85.9%
Hyperactivity	86%
Inability to sit still where they are expected to	86.7%
Often has difficulty organizing tasks and activities	79.3%
Need to monitor them while doing their homework	92.7%
They only pay attention to what they are highly interested in	88%
Reluctance to do their homework	84.7%
Restlessness and fidgetiness	90%
Difficulty in waiting their turn	79.9%
Inability to play quietly	73.3%

Less than one third of parents believed that this disorder is hereditary. More than one third of the parents believed that bad parenting caused the disorder. In a study in 2007 in Shiraz (15), 52% and 47.7% of parents attributed ADHD to bad parenting and genetic and biologic vulnerabilities, respectively. In a study by Pham et al., in 2010, 40% of the parents believed that the main cause of ADHD is a chemical imbalance in the brain (16). Ascribing ADHD to bad parenting may develop feeling of guilt in the parents, as well as parental conflict, so its modification reduces such feelings (17). Of the parents, 62% did not attribute the disorder to high IQ; similar results were obtained in other studies (15). More than half of the parents believed that diagnosis requires specific psychological tests, while it is based on clinical interview with parents and children (1). Over 70% of the parents believed that intense activity consumes children energy and consequently reduces their symptoms, and they view this as a neurobiological disorder that is associated with inhibitory control problems (18) and inability to delay gratification (19). However, ADHD is not due to high energy level, and thus extra activity and severe fatigue do not reduce the symptoms. Despite the fact that ADHD treatment mainly includes the administration of stimulants and behavioral interventions (20), less than half of the parents believed in giving medication to their children. This is because they may not consider ADHD a disease, and they reject pharmacotherapy. In a study in the USA, 16.8%, 24.4%, and 53.8% of the parents supported the mere administration of pharmacotherapy, non-medicinal treatments, and a combination of both to control the disorder, respectively (16). Other research also indicate negative parental attitude towards using psychotropic drugs (21). Of the parents, 25% associated Ritalin with serious and significant complications which is an important factor for their unwillingness towards using this medicine, while the effectiveness and safety of stimulants have been proven in many studies (22). Among the parents, 75% did not believe in disorder improvement by aging, and other studies have demonstrated disorder continuance to adulthood in 40-70% of the cases (23). In a study by Ghanizadeh et al., almost all parents rejected spontaneous improvement of the disorder and highlighted the need for treatment. Moreover, a small percentage of parents believed that this is a lifetime disorder. In this study, parents reported average improvement age of 16.5 (ranging from 12-21) (15). This false belief is also another barrier against treatment. One-third of the parents noted that the lack of treatment did not result in more problems, and over 40% assumed that substance abuse and other psychological problems increased with ADHD. However, prognosis in these patients is related to the length of treatment (24). In a study by Ghanizadeh, over half of the parents believed that this disorder was associated with the risk of delinquency. In addition, two-third of the parents assumed ADHD as a serious problem (15). In this study, television was the main source of information (60%). In another research in Shiraz, radio and television were the source of information in 74.9% of the cases. Although 88% of the parents were familiar with ADHD prior to the study, their information was not adequate and appropriate, especially with respect to the etiology, course and treatment. The authorities should pay special attention to provide proper and comprehensive warning about ADHD to the parents through media. Undoubtedly, the lack of attention or limited information of other physicians, especially general practitioners (GP) and pediatricians, about ADHD has expanded the role of media to the main source of information in this area. In a study in 2010 in Shiraz, less than half of GPs considered themselves adequately knowledgeable about this disorder (25). A study in Pakistan and Turkey (26, 27) showed similar results. Given that these physicians are more connected with children, expanding their knowledge about ADHD seem essential. 

In addition, teachers of one-fifth of these children did not recognize their problem. In another study by Ghanizadeh in 2006, Iranian teachers' awareness of ADHD has been reported to be low (15). 

In a study in Rasht, the majority of primary school teachers had moderate knowledge about ADHD (28). Similar studies in other countries have provided different results (29). Given that teachers are in direct connection with educational and behavioral matters of children, they are required to obtain full knowledge about it. This empowers them to detect children with ADHD, refer them to a physician and appropriately manage them in the class. Therefore, training teachers more in this area is of prime importance.

## Conclusion

In general, results of this study highlight the importance of informing parents about the symptoms, etiology, prognosis and treatment of ADHD. Increasing parents' knowledge and correcting their myths decrease treatment resistance, leading to ADHD prognosis improvement and complications decrease (11).

## Limitations

This study had some limitations. First, the participating parents themselves detected a problem in their children and asked for an evaluation; therefore, they were probably more knowledgeable than parents in the general population. Second, information level may differ in various therapeutic phases. Therefore performing studies on the general population with larger sample size is required. Furthermore conducting an interventional study is recommended in this area, in which parents' knowledge and attitude are investigated and compared, before and after the provision of a training package. Another limitation was that the study was conducted in one center; therefore, the participants may have the same social class, so the results could not be generalized to the general population, and community sample studies are necessary.
